# Early Cretaceous sea surface temperature evolution in subtropical shallow seas

**DOI:** 10.1038/s41598-021-99094-2

**Published:** 2021-10-05

**Authors:** Stefan Huck, Ulrich Heimhofer

**Affiliations:** Institut für Geologie, Leibniz Universität Hannover, Callinstraße 30, 30167 Hannover, Germany

**Keywords:** Geochemistry, Palaeoclimate

## Abstract

Late Cretaceous sea surface temperatures (SST) are, amongst others, traditionally reconstructed by compiling oxygen isotope records of planktonic foraminifera obtained from globally distributed pelagic IODP drill cores. In contrast, the evolution of Early Cretaceous SSTs is essentially based on the organic TEX_86_ palaeothermometer, as oxygen-isotope data derived from well-preserved ‘glassy’ foraminifer calcite are currently lacking. In order to evaluate the extraordinary warm TEX_86_-derived SSTs of the Barremian to Aptian (130–123 Ma) subtropics, we present highly resolved sclerochemical profiles of pristine rudist bivalve shells from Tethyan and proto-North Atlantic shallow water carbonate platforms. An inverse correlation of seasonal ontogenetic variations in δ^18^O_rudist_ and Mg/Ca ratios demonstrates the fidelity of oxygen isotopes as palaeotemperature proxy. The new data shows moderate mean annual SSTs (22–26 °C) for large parts of the Barremian and Aptian and transient warm pulses for the so-called Mid-Barremian Event and Oceanic Anoxic Event 1a (reaching mean annual SSTs of 28 to 30 °C). A positive shift in mean annual oxygen-isotope values (δ^18^O: ≤ − 0.3‰) coupled with invariant Mg/Ca ratios at the Barremian–Aptian boundary points to a significant net loss of ^16^O in Tethyan shallow-marine settings. As the positive oxygen-isotope rudist shell values are recorded immediately beneath a major superregional hiatal surface, they are interpreted to be related to a major cooling phase and potential glacio-eustatic sea-level lowering. Our new sclerochemical findings are in clear contrast to open ocean SST records based on TEX_86_, which indicate exceptionally warm Barremian to earliest Aptian subtropical oceans and weak meridional SST gradients.

## Introduction

The study of past greenhouse climates such as the Cretaceous provides fundamental insights into Earth’s response to increased concentrations of greenhouse gases. In this context, proxy data-based paleoenvironmental reconstructions play a central role in evaluating the ability of climate models to simulate past, present and future climate change^1^. In particular, the assessment of stratigraphically well-constrained high-resolution temperature (and CO_2_) proxy records is paramount for a better understanding of the potential range and rate of future climate change^2^.

The Cretaceous climate is generally described as a warm to hot greenhouse world characterized by high atmospheric CO_2_ levels and weak equator-to-pole thermal gradients (e.g.,^3,4^). The Early Cretaceous climatic warming culminated in the Cenomanian–Turonian Thermal Maximum and was followed by a gradual long-term cooling trend towards the Early Maastrichtian ‘cool’ greenhouse (e.g.,^5–7^). The stability of the greenhouse climate has long been questioned by a variety of sedimentological, palaeontological, geochemical, sequence stratigraphic and modelling data. In fact, there is abundant evidence for the existence of Cretaceous ‘cold snaps’ or the potential transient occurrence of polar ice sheets (e.g.,^8–16^).

One of the most important diagnostic features for earth’ climate state and its variability (in deep time) is sea surface temperature (SST). Reconstructions of Cretaceous open ocean SSTs are predominantly based either on crenarchaeotal membrane lipid distributions (isoGDGTs) from pelagic deposits (referred to as TEX_86_ proxy) or on oxygen isotope compositions recorded in low-Mg calcite hard parts of planktonic foraminifers^4,6,7,16–22^. Late Cretaceous oxygen isotope and TEX_86_ SST records provided by ocean drilling campaigns are usually highly resolved and stratigraphically well-calibrated, which (1) provides a very detailed record of SST change with time across paleolatitude and (2) allows for a comparison with deep-sea δ^18^O trends as recorded in benthic foraminiferal calcite^5^. In contrast, Early Cretaceous pelagic deposits lack well-preserved (glassy) planktonic foraminifer tests, which in turn hampers any critical evaluation of the extraordinary warm Early Cretaceous TEX_86_ SSTs (> 35 °C)^6^. Alternative substrates for oxygen isotope palaeothermometry are the calcitic guards of belemnites^23,24^. Due to the nektonic lifestyle of these squid-like organisms, however, belemnite-based SST records are likely influenced by differences in habitat depths ranging from 200 m towards the sea surface^25^.

Complementary to pelagic proxy records, rudist bivalve shells have been proven as suitable shallow marine neritic SST archive^26–28^. Their thick low-Mg calcite shells allow for a quantitative assessment of ontogenetic oxygen isotope and Mg/Ca variations at a sub-annual (seasonal) resolution^29,30^—referred to as chemical sclerochronology^31^.

A SST dataset covering pre-Albian times with a similar stratigraphic precision to pelagic TEX_86_ records is currently not available. Therefore, high-resolution chemical sclerochronology (δ^18^O, Mg/Ca, Sr/Ca) has been performed on pristine (i.e., compact and fibrous, and chemostratigraphically well-constrained) rudist shells collected from subtropical Barremian–Aptian shallow-marine carbonate platform sections. The overall aim is to (1) reconstruct the Barremian–Aptian evolution of subtropical mean annual SSTs as well as their seasonal variability. Of particular interest is (2) the contrast comparison of rudist shell-derived SST estimates with existing TEX_86_ as well as belemnite δ^18^O compilations and the (3) detection of climate extremes (hyperthermals, cold snaps) within the greenhouse state.

## Palaeogeographic and palaeoenvironmental setting

**Figure 1:**
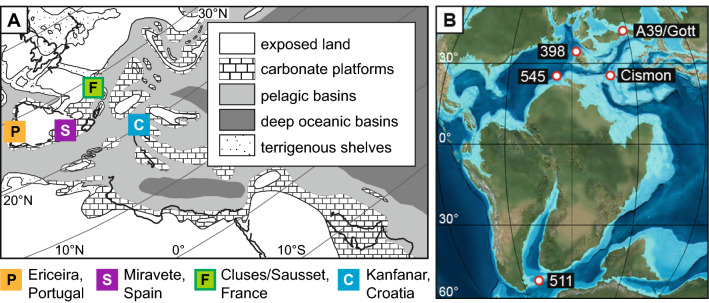
(**A**) Early Aptian paleogeography of the Atlantic-Tethyan realm with localities of considered study sites in Portugal (Ericeira), Spain (Miravete), France (Sausset and Cluses) and Croatia (Kanfanar section). Map modified after Steuber et al.^27^. (**B**) Aptian paleogeography of the Tethyan and proto-North-Atlantic realm with locations of considered pelagic sections and oceanic drill sites. Map modified from R. Blakey, https://deeptimemaps.com/.

Rudist bivalves were collected from five Barremian to Aptian carbonate platform sections (Fig. 1A; Ericeira, Portugal = P, Miravete, Spain = S, Sausset and Cluses, France = F, Kanfanar, Croatia = C) representing subtropical Tethyan and proto-North Atlantic shallow water settings between 23° and 32° N. Paleolatitude estimates of the different localities are based on van Hinsbergen et al.^32^.

Rudists belong to the sessile benthos and all studied taxa (Requienidae, Polyconitidae, Monopleuridea) are considered to have inhabited an inner carbonate platform domain with negligible meteoric influence^33^. The pristine preservation state of all analyzed shells is proven by means of elemental and stable isotope properties (see SI Appendix Dataset S1) and subordinately cathodoluminescence characteristics^34,35^. Age assignments of shells are based on an integrated carbon and strontium isotope stratigraphic framework (Fig. 3:^34–37^; see “Material and methods” section).

## Results

**Figure 2:**
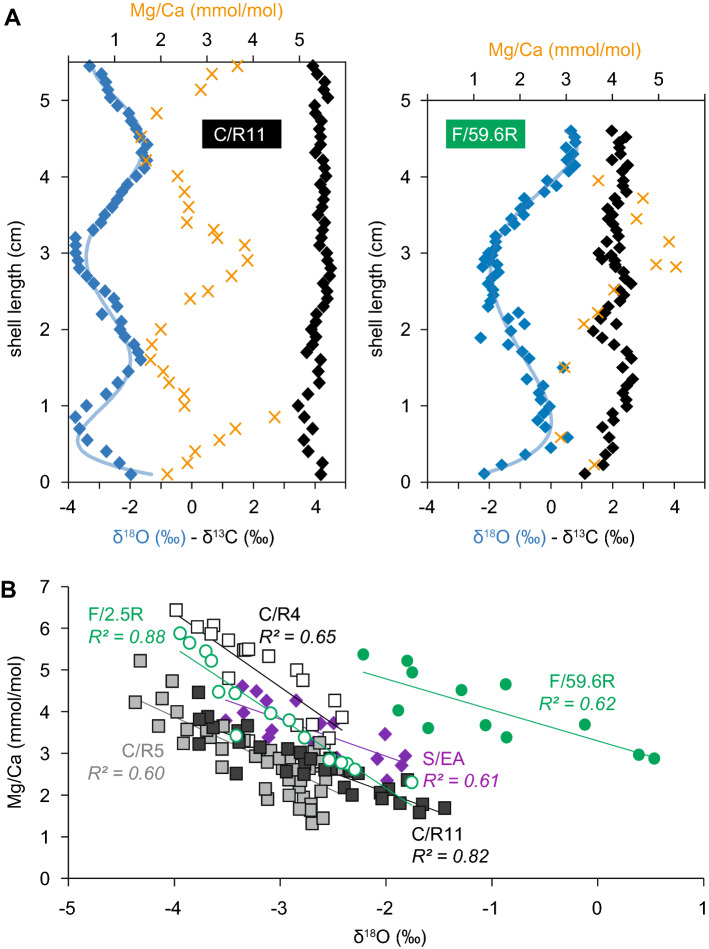
(**A**) Sclerochemical results (δ^18^O, δ^13^C, Mg/Ca) of selected rudist shells from Croatia (C/R11) and France (F-59.6R). Blue lines represent polynomial trendlines. (**B**) Cross-plot of δ^18^O_rudist_ and Mg/Ca molar ratios selected rudist shells from France (F/2.5; F/59.6), Croatia (C/R4; C/R5; C/R11) and Spain (S/EA).

**Figure 3:**
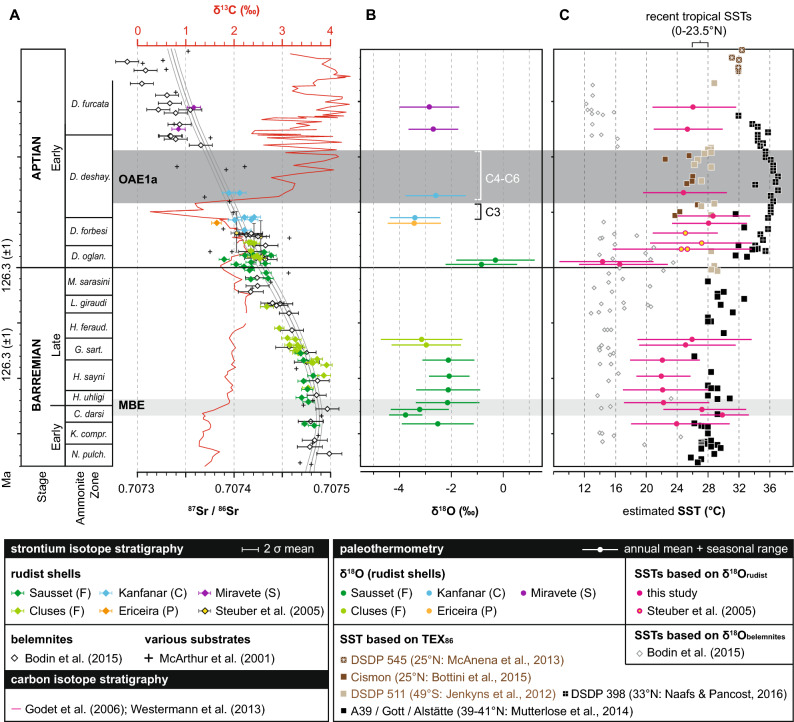
(**A**) Evolution of Barremian–Aptian global marine ^87^Sr/^86^Sr and carbon-isotope ratios compiled from previous work on pelagic carbonate bulk and low-Mg calcite fossils (belemnites, bivalves). (**B**) Oxygen isotope sclerochronology of selected rudist shells—this study. (**C**) Sea surface temperature (SST) estimates based on oxygen isotope palaeothermometry (belemnites, rudists) and TEX_86_. Note that rudist sclerochronology also provides information on the seasonal range of SSTs. All results are calibrated to the GTS 2012 timescale^75^. See figure for references.

Most shells record cyclic, often sinusoidal oxygen-isotope variations with peak-to peak amplitudes ranging between 1.6 and 3.1‰ (Figs. 2A, 3, see also SI Appendix, Figures S1–S3 and Dataset S1 for detailed results). Annual growth rates depicted by the wave-lengths of these (seasonal) δ^18^O cycles vary from 2 to 6 cm.

Rudists from the Provence and Jura-Bas-Dauphiné platforms in SE France (Sausset/Cluses: 29°–32° N) provide sclerochronological mean oxygen isotope (δ^18^O_sclero_) values ranging between − 3.9 (uppermost Lower Barremian) and − 0.3‰ (lowermost Lower Aptian). The majority of upper Barremian δ^18^O_sclero_ values from the Provence cluster around a value of − 2.1‰. Lower Aptian (pre-OAE1a) rudists inhabiting both proto-Atlantic (Ericeira: 27° N) and Tethyan (Kanfanar: 23° N) shallow-water settings show δ^18^O_sclero_ values near to − 3.4‰. At Kanfanar, rudist shell material ascribed to the onset of OAE1a (chemostratigraphic segment C4)^38^ provides a δ^18^O_sclero_ value of − 2.6‰. Slightly lower δ^18^O_sclero_ values (− 2.8‰) characterize post-OAE1a rudist shells from E Spain (Miravete: 26° N).

Shells are characterized by low Fe (mean: 43 ppm; s.d.: 40 ppm) and Mn contents (mean: 3 ppm; s.d.: 7 ppm) and high Sr contents (mean: 1140 ppm; s.d.: 122 ppm). Detailed element compositions (ppm) and magnesium/calcium ratios are presented in SI Appendix, Dataset S1. Here, we only report on Mg/Ca molar ratios, which are—at least in a qualitative manner—assumed to be linked to sea surface temperature^39^. Mean Mg/Ca ratios of sclerochronological shell transects range between 2.7 (C/R11) and 4.9 (C/R4). All analyzed shells provide evidence for a cyclic Mg/Ca pattern, with peak-to-peak amplitudes varying from 2.5 (sample F/59.6R; Fig. 2A) to 3.6 (sample F/2.5R). Mg/Ca cycles are very well anti-correlated with oxygen-isotope cycles (e.g., S/EA: r = − 0.78; *p* < 0.001; C/R11: r = − 0.91; *p* < 0.001) (Fig. 2B and SI Appendix, Dataset S1).

## Discussion

### Fidelity of rudist shell elemental and stable isotope compositions

Polished slabs document the pristine preservation state of analyzed rudist shells by the occurrence of low-Mg calcite fibers arranged in well-distinguishable growth increments (SI Appendix, Figures S1 and S3). Measured elemental (Mg, Sr, Fe, Mn) and isotope compositions (δ^18^O, δ^13^C) overlap very well with those assessed from diagenetically screened Cretaceous rudist shells collected from similar latitudinal settings^29^. ^87^Sr/^86^Sr values (Fig. 3) are in excellent agreement with Barremian–Aptian marine strontium-isotope compilations^40^. The fidelity of both, stable isotope and elemental shell data is emphasized by a cyclic sclerochronological pattern of both oxygen-isotope and Mg/Ca molar ratios (Fig. 2A). Only few stable isotope outliers escaped the diagenetic screening protocol.

Observed asymmetric δ^18^O cycles typically reflect variable intra-annual shell growth rates, i.e. relatively enhanced growth rates during the colder seasons (e.g., sample C/R1: SI Appendix, Dataset S1). Occasionally occurring saw-tooth shaped peaks (e.g., sample F/62.3R: SI Appendix, Dataset S1) indicate growth cessations associated with seasonal (here: cool) SST extremes^41,42^. A well-expressed anti-correlation of δ^18^O and Mg/Ca molar ratios (Fig. 2B) corroborates the application of oxygen isotopes as palaeotemperature proxy, although a multitude of studies (e.g., 43) on recent and fossil bivalves provide evidence that other factors (e.g., metabolic activity, growth rate, ontogenetic age) influencing the Mg/Ca molar ratio of individual shells have to be considered. The observation of different slopes of δ^18^O-Mg/Ca regression lines (Fig. 2B, see also SI Appendix, Figure S4), provided by different taxa (*Toucasia carinata, Requienia zlatarskii*: C-R4 and C-R5) collected from the same stratigraphic level, demonstrates a species-specific biological control on recorded Mg/Ca ratios^30,43^.

### Barremian–Aptian neritic SST evolution

Rudist shell δ^18^O values provide evidence for a transient (< 400 kyr) warming pulse (27–30 °C) associated with the Mid-Barremian Event (MBE; *Coronites darsi* ammonite zone)^35^. In the Tethyan Ocean, the MBE is characterized by a 0.5‰ positive carbon-isotope shift and the onset of black-shale deposition^44^. Low-Mg calcite belemnite guards from the boreal realm (A39 and Gott sections: Fig. 1B) document a similar climatic scenario (SST increase from 16 °C towards 20 °C), which is referred to as *Aulacoteuthis* warm pulse^45^. Belemnites from the Vocontian Basin, in contrast, do not provide evidence for a pronounced negative oxygen isotope excursion during the MBE^46^. There, Barremian transient negative δ^18^O shifts in belemnite calcite in the prelude and aftermath of MBE were proposed to be caused by the impact of meteoric water^46^. The circum-Vocontian rudist-bearing carbonate platform sections studied here (Sausset, Cluses:^34,35^), however, do not provide any paleobiological evidence (e.g., reduced biodiversity, lack of stenohaline organisms or spread of charophyceans or microbial mats) for a significant salinity reduction (7–8 PSU considering a 1.3–1.6‰ δ^18^O_sw_ change) due to freshwater inflow^29,47^.

Relatively temperate climatic conditions with mean annual SSTs of about 22 °C prevail during the early Late Barremian (*Hyperaspis uhligi* and *Heinzia sayni* ammonite zones), followed by a mid-Late Barremian SST rise towards 26 °C (*Gerhardtia sartousiana* ammonite zone) (Fig. 3). Upper Barremian belemnites both from the boreal and Tethyan realms document relatively cool temperatures^46^, which are significantly lower (14–16 °C) than seasonal SST minima (17–19 °C) of contemporaneous rudist shells. As mentioned earlier, this difference is likely explained by differences in habitat depth^25^. Remarkably cool mean annual SSTs (14 and 16 °C) are derived from lowermost Aptian rudist shells (*Deshayesites oglanlensis* ammonite zone). Additional proxy-based evidence for a transient earliest Aptian episode of climatic cooling comes from fish teeth apatite palaeothermometry (amplitude: 4–6 °C)^48^. Strongly declining rudist shell oxygen isotope values in the prelude of the Early Aptian OAE1a provide evidence for rapid climatic warming, reaching maximum mean annual SSTs of about 28 °C at the onset of the event (*Deshayesites forbesi* ammonite zone, chemostratigraphic segment C3 of^38^). During OAE1a (chemostratigraphic segment C4: *Deshayesites deshayesi* ammonite zone) rudist mean annual SSTs decline by about 3 °C (Fig. 3). This cooling episode has been previously noticed by a high-resolution oxygen-isotope study carried out on well-preserved pelagic nannofossil-rich carbonate bulk material^49^ and is compatible with an atmospheric drawdown of CO_2_ due to enhanced organic carbon burial and silicate weathering^50^. In the aftermath of OAE1a (*Deshayesites furcata* ammonite zone), rudist shell mean annual SSTs prevail at about 25–26 °C. Again, belemnites provide relatively constant and significantly cooler Aptian SSTs^46^, an observation that may be explained both by the habitat depth and migration pattern of these free-swimming organisms (see^25^ for a discussion).

### Comparison with open ocean SST estimates provided by TEX_86_

Early Cretaceous SST compilations based on the TEX_86_ paleothermometer show rather stable and warm Barremian SSTs (Early Barremian: 26–30 °C; Late Barremian: 26–32 °C) and a switch to extraordinary warm Aptian SSTs (> 35 °C). Interestingly, the majority of TEX_86_ SST estimates overlap with or even exceed seasonal SST maxima (26–34 °C) identified in rudist shell calcite (Fig. 3). This observation is in concert with the well-documented offset between Late Cretaceous TEX_86_ and planktonic δ^18^O SSTs and points to a seasonally biased TEX_86_ SST signal^51,52^. In fact, the maximum abundance of Thaumarchaeota has been reported to vary both with seasonality and depth depending on the locality studied (see^21^ for a review). Only two sites (Fig. 1B) representing both, a low latitude Tethyan (Cismon section; 25° N:^53^) and a middle latitude southern Atlantic setting (DSDP 511; 49° S:^54^) provide Early Aptian TEX_86_ SSTs (24–28 °C) that agree with contemporaneous sclerochronological mean annual SST values (Fig. 3), but deviate significantly from SSTs (> 35 °C) provided by other TEX_86_ proxy records (DSDP 398; 33° N:^55^). Following Steinig et al.^56^, these observed differences in TEX_86_ SSTs might be caused by regionally different TEX_86_-temperature calibrations that either reduce (DSDP 398) or increase (Cismon) SST estimates.

The new rudist shell-based sclerochronological records are therefore essential to (1) record stratigraphically well-constrained and reliable SSTs for the late Early Cretaceous subtropics and to (2) evaluate highly-resolved TEX_86_ SST compilations reflecting various localities that show potential regional deviations of the TEX_86_-temperature relation from current global core-top calibrations^55^.

### Evaluating SST seasonality during a phase of climate instability

Most of the Barremian–Aptian shells under study provide relatively enhanced intra-shell δ^18^O peak-to-peak amplitudes, with mean values ranging between 2.1 (Early-early Late Barremian; Early-Late Aptian) and 2.9‰ (Late Barremian, early Early Aptian). Without considering intra-annual salinity changes, these δ^18^O peak-to-peak amplitudes would translate into subtropical SST seasonalities ranging between 6.7 (onset of MBE warming) and 13.7 °C (Late Barremian). Rudists with sclerochronological evidence for a Barremian–Aptian cooling event provide relatively enhanced seasonal SST ranges (< 12.2 °C). This is surprising, as during the present-day icehouse stage characterized by enhanced equator-to-pole SST gradients, seasonal SST amplitudes recorded in subtropical settings are considerably lower (8.5 °C: e.g., Northern Bahamas, 26.7° N, 78.4° W)^57^. One explanation for the in general high SST seasonalities would be that recorded seasonal δ^18^O patterns also reflect local salinity-controlled δ^18^O_SW_ changes. In a previous sclerochronological study on a Late Cretaceous subtropical elevator rudist^58^, the authors pointed out that the recorded seasonal amplitude of 11 °C maybe overestimated by up to 7.5 °C due to a seasonal δ^18^Osw fluctuation of up to 1.5‰. Considering the paleogeographic position of the here studied Northern Tethyan and proto-North Atlantic rudist-bearing sections in the subtropical arid belt, significant seasonal δ^18^O_SW_ variations (> 1‰) are unlikely^27^.

### Exceptionally high δ^18^O values above the Barremian–Aptian boundary

Cross-plots of δ^18^O_rudist_ and Mg/Ca (molar) ratios provide evidence for a strong temperature effect on the incorporation of both oxygen isotopes and Mg into rudist shell calcite. The observation of species and specimen specific regression lines (Fig. 2B) is in line with Steuber and Rauch^30^. One exception is a lowermost Aptian rudist shell collected from the Provence carbonate platform (F/59.6R), which shows a similar regression line slope but significantly more positive δ^18^O values (see also^35^). If δ^18^O_rudist_ values are considered to solely represent SSTs, the observed abrupt positive shift would translate into a major subtropical cooling event at around the Barremian–Aptian boundary that is characterized by a temperature drop of about 8 °C (Fig. 3). Apart from fish teeth apatite^48^ recording a synchronous but less pronounced SST fall in the order of about 4 °C, additional proxy-based evidence for this cooling event is currently lacking. The amplitude of the here documented SST shift as indicated by strongly enhanced δ^18^O_rudist_ values, however, might be overestimated if locally or globally acting processes such as enhanced evaporation preferentially removing ^16^O or alternatively, enhanced removing and storage of ^16^O in ephemeral polar and/or continental ice are considered. A strongly evaporative setting is unlikely, however, as it is expected to result in significant biotic changes (e.g., monospecific assemblages of biota adapted to hypersaline conditions), which haven’t been observed in the rudist bearing platform carbonates^35,59^. The possible existence of Early Cretaceous polar ice has been debated for decades (e.g.,^10^). Modelling studies show that ice growth during the Aptian might have been possible^60^. Indeed, Al-Husseini et al.^61^ interpreted the well documented and widespread loss of shallow water carbonate platforms at the Barremian–Aptian boundary as indirect evidence for a short term (~ 800 kyr) glaciation event, although the proposed sea-level fall (> 30 m) does not mutually exclude other factors. Still, the difference between the observed positive oxygen-isotope anomaly and pre-OAE1a values of about 2‰ is hard to explain simply by ice growth, if compared with the glacial–interglacial change in deep-sea δ^18^O of about 1‰ since the (Pleistocene) Last Glacial Maximum^62^. Modelled Cretaceous sea surface water oxygen-isotope maps^63^, however, nicely illustrate the variability of δ^18^Osw in the Tethyan and proto-North Atlantic shallow water realm, which is also governed by the local paleogeographic and bathymetric setting.

In order to evaluate the cooling episode postulated here and the potential growth of ice sheets around the Barremian–Aptian boundary, additional sclerochemical results from stratigraphically well-constrained carbonate platform sections are essential. If available, these (seasonally resolved) data will allow a unique insight into a tipping point of the Cretaceous climatic evolution, in particular if combined with additional independent palaeotemperature proxies such as clumped isotopes. The latter proxy will furthermore allow reconstructing seasonal δ^18^O_SW_ variations and thereby testing the seasonal range of SSTs depicted by δ^18^O values in rudist shells.

## Material and methods

### Sclerochemistry

The outer fibrous low-Mg calcite shell layers of 23 well-preserved rudists were analyzed for ontogenetic variations in δ^18^O, δ^13^C and major and trace element (Ca, Mg, Sr, Fe and Mn) contents. Sclerochronological sampling along cross sections (number of subsamples: 13 to 80; mean: 40; s.d.: 17) followed the maximum growth axis of the shell. As elemental geochemistry requires a relatively large amount of powdered carbonate, some sclerochronological profiles could only be sampled at a relatively low resolution. As these low-resolution profiles would have likely captured a lower seasonal range of δ^18^O_rudist_ values, additional high-resolution profiles of contemporaneous shells were produced. Occasionally, the limited size of analyzed shell transects hampered the assessment of more than one δ^18^O cycle. In this case, sclerochronological profiles of several rudist fragments derived from the same stratigraphic level were compiled in order to faithfully evaluate seasonal δ^18^O patterns. In general, only oxygen isotope values of identified individual seasonal δ^18^O cycles were used to calculate sclerochronological mean, minimal and maximal values.

Carbonate powder samples were extracted from carbonate slabs by means of a hand-held PROXXON IBS/E drill equipped with tungsten carbide drill bits. In order to avoid shell portions that are affected by bioerosion or diagenetic calcite material, sampling was performed under a binocular microscope. Stable isotope analysis of 892 samples was performed at the isotope laboratory of the Institute of Geology at Leibniz University Hannover, Germany, using a Thermo Fisher Scientific Gasbench II carbonate device connected to a Thermo Fisher Scientific Delta 5 Advantage isotope ratio mass spectrometer. Samples are treated with viscous water-free (98 g mol^−1^) orthophosphoric acid at 72 °C to release CO_2_ of the calcite 1 h before the start of the measurement. Repeated analyses of certified carbonate standards (National Bureau of Standards (NBS) 19: δ^13^C/δ^18^O = 1.95/− 2.2; International Atomic Energy Agency (IAEA) CO-1: δ^13^C/δ^18^O = 2.492/− 2.4; IAEA CO-8: δ^13^C/δ^18^O = − 5.764/− 22.71) show an external reproducibility (standard deviation) of ≤ 0.06‰ for δ^13^C and ≤ 0.08‰ for δ^18^O. Values are expressed in conventional delta notation relative to the Vienna-Pee Dee Formation belemnite (VPDB) international standard, in parts per mil (‰).

$$\updelta ^{18} {\text{O}} = \left( {^{18} {\text{O/}}^{16} {\text{O}}_{{{\text{sample}}}} {/}^{18} {\text{O/}}^{16} {\text{O}}_{{{\text{standard}}}} {-} \, 1} \right)*1000$$

Aliquots of 142 powdered rudist samples (1.35–1.65 mg) were analyzed for their elemental composition using inductively coupled plasma-atomic emission spectrometry (ICP-AES) at the isotope laboratory of the Institute of Geology, Mineralogy and Geophysics at Ruhr-University Bochum (RUB), Germany. Selected samples with low manganese concentrations (threshold value of < 100 ppm) and high strontium concentrations (threshold value of > 800 ppm) were analyzed at RUB for their strontium-isotope ratios (n = 5) using a thermal ionization mass-spectrometer (Finnigan MAT 262) in dynamic mode. Corrections of measured strontium-isotope ratios to a USGS EN-1 value of 0.709175 (rather than to NIST SRM987) were done following the procedure of Howarth and McArthur^64^.

### Oxygen isotope palaeothermometry

Oxygen isotope palaeothermometry builds on the temperature-dependent oxygen isotope fractionation between CaCO_3_ and water^65–68^. Its application to (marine) bivalve shells requires knowledge of the oxygen isotope composition of the carbonate precipitating body fluid, which is thought to be in equilibrium with ambient sea water (δ^18^Osw)^68^. In order to account for latitudinal differences in evaporation and precipitation (i.e., salinity) leading to substantial meridional δ^18^O_SW_ gradients^63,69–71^, δ^18^O_SW_ values were adjusted to salinity following the modelled mid-Cretaceous latitudinal δ^18^O_SW_ distribution of Poulsen et al.^69^. Sea surface temperatures (SSTs) are calculated by using the equation of Anderson and Arthur^72^.

$${\text{T}}\;^\circ {\text{C}} = 16.0 - 4.14*\left( {\updelta ^{18} {\text{O}}_{{{\text{calcite}}}} -\updelta ^{18} {\text{O}}_{{{\text{SW}}}} } \right) + 0.13*\left( {\updelta ^{18} {\text{O}}_{{{\text{caclite}}}} -\updelta ^{18} {\text{O}}_{{{\text{SW}}}} } \right)^{2}$$

δ^18^O_SW_ values range between − 0.91 and − 0.55‰ and thus produce SST estimates that are 0.4–2.1 °C warmer than those based on a uniform δ^18^O_SW_ (SMOW) of − 1‰^73^.

### Stratigraphic framework

The integrated strontium and carbon isotope stratigraphic framework presented here is compiled from previous work^34–37^ and in part complemented by additional strontium isotope measurements. We do not transfer ^87^Sr/^86^Sr values recorded by rudist shells into numerical ages based on the look-up table (version 4: 08/2003) of McArthur et al.^40^, as the LOWESS curve is a bimodal fit of ^87^Sr/^86^Sr values derived from a lot of different sources and environments. In contrast, superimposed stratigraphic trends of ^87^Sr/^86^Sr values and biostratigraphic markers are used to identify a stratigraphic age range. Subsequently, a more robust time control and outstanding resolution is achieved by a carbon-isotope chemostratigraphic correlation of considered rudist-bearing shallow water sections with Tethyan pelagic successions (GTS2012 time scale)^74–76^ (SI Appendix, Figures S1–S4).

## Supplementary Information

Supplementary Figure S1.

Supplementary Figure S2.

Supplementary Figure S3.

Supplementary Figure S4.

Supplementary Information.

Supplementary Legends.
