# Supplementary material for: Early Cretaceous sea surface temperature evolution in subtropical shallow seas
Source: Sci Rep. 2021 Oct 5;11:19765. doi: 10.1038/s41598-021-99094-2 (PMC8492702; doi:10.1038/s41598-021-99094-2)
Supplement: Supplementary file 1 — Supplementary Figure S1. [file 41598_2021_99094_MOESM1_ESM.pdf]

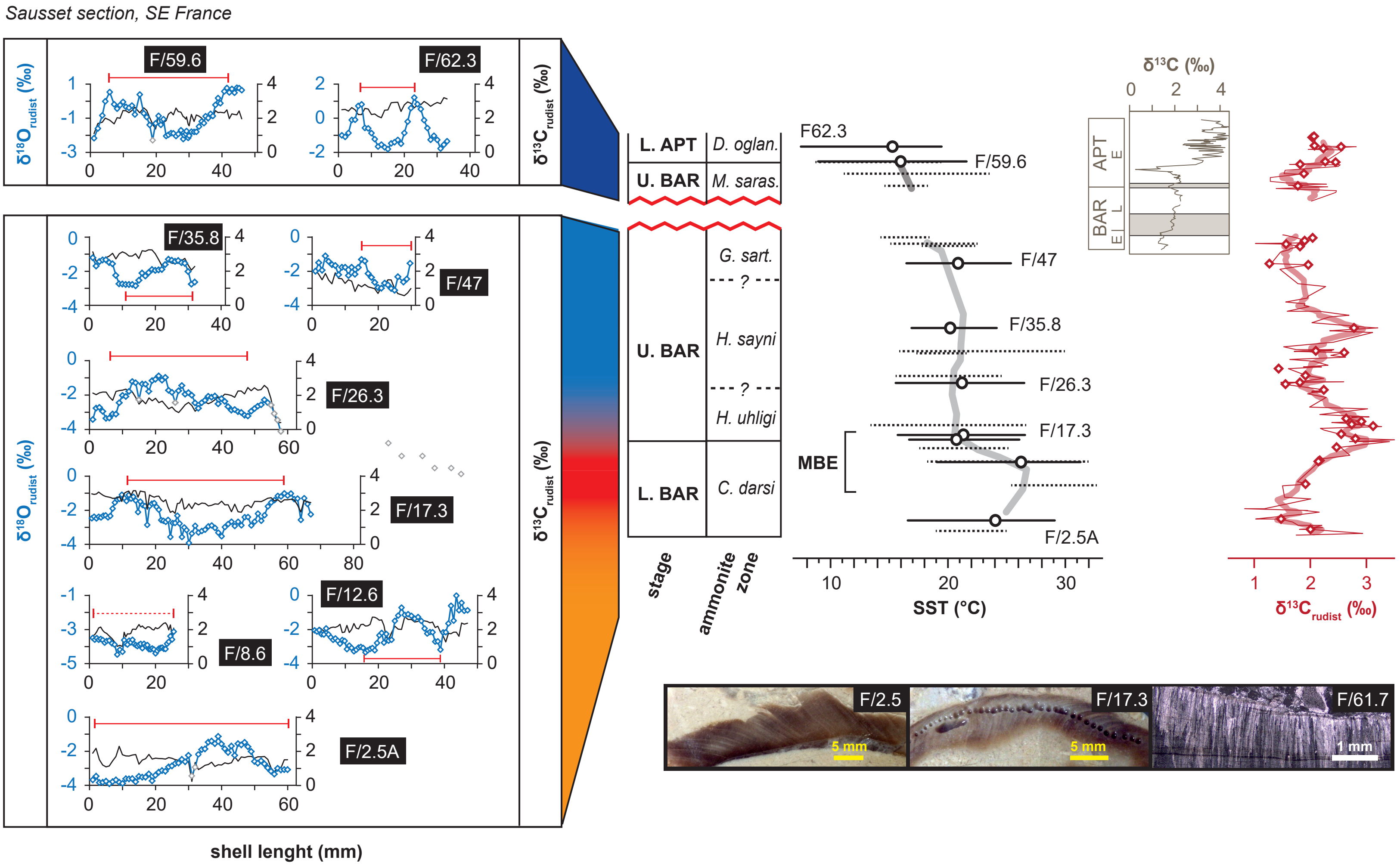
Sausset section, SE France
F/59.6
F/62.3
δ18Orudist (‰)
δ13Crudist (‰)
F/35.8
F/47
F/26.3
F/17.3
F/12.6
F/8.6
F/2.5A
shell lenght (mm)
L. APT
D. oglan.
U. BAR
M. saras.
G. sart.
H. sayni
H. uhligi
L. BAR
C. darsi
stage
ammonite zone
F62.3
MBE
SST (°C)
δ13C (‰)
APT
BAR
F/2.5
F/61.7
5 mm
1 mm
