# Supplementary figures and images for: Early Cretaceous sea surface temperature evolution in subtropical shallow seas
Source: Sci Rep. 2021 Oct 5;11:19765. doi: 10.1038/s41598-021-99094-2 (PMC8492702; doi:10.1038/s41598-021-99094-2)

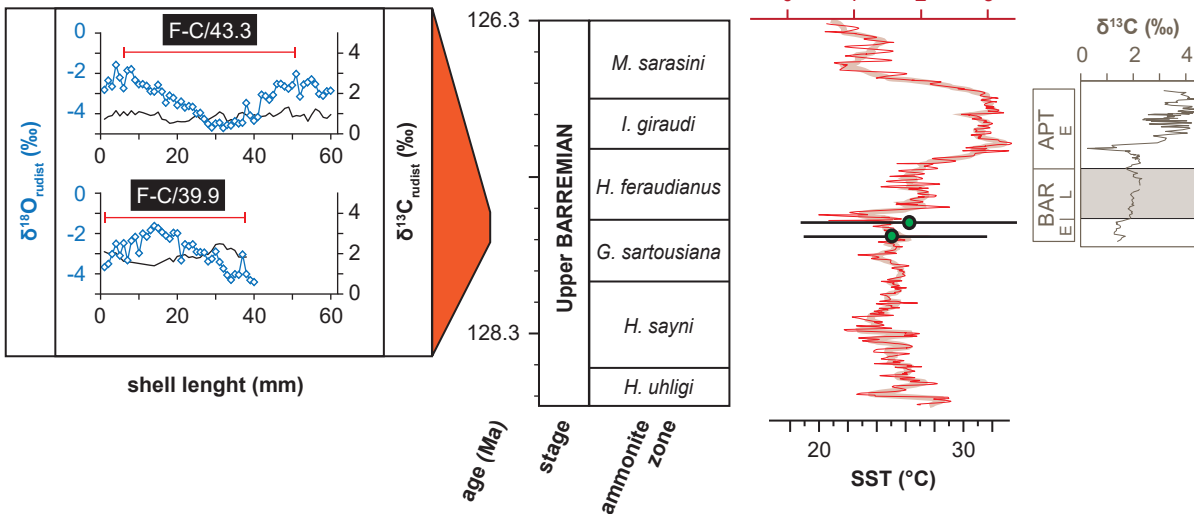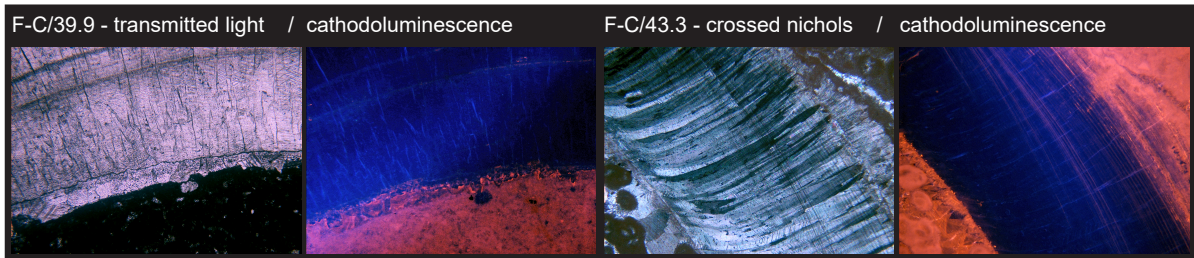

Supplement: Supplementary file 2 — Supplementary Figure S2. [file 41598_2021_99094_MOESM2_ESM.pdf]

Kanfanas section (KAN) - Croatia

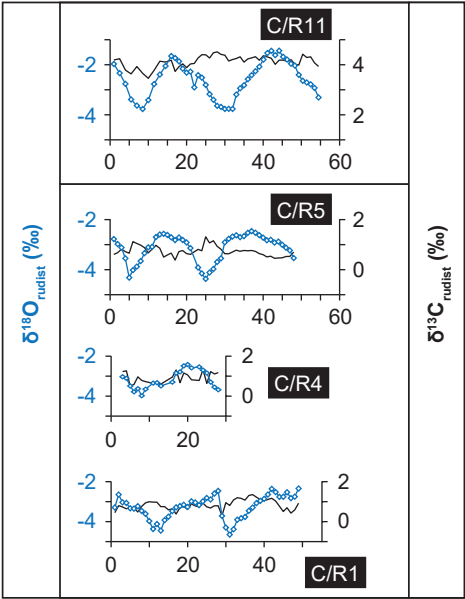

shell length (mm)

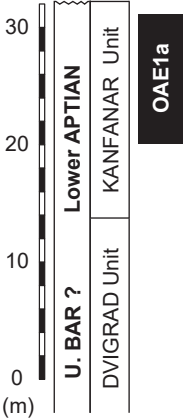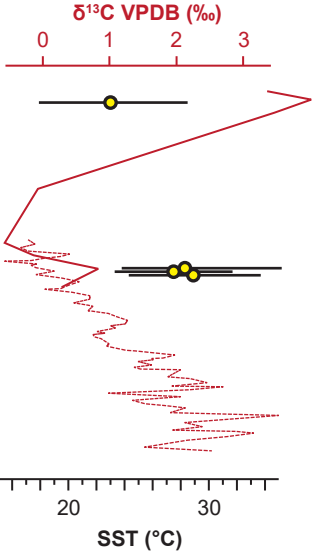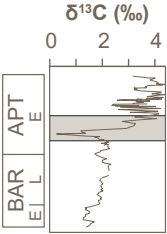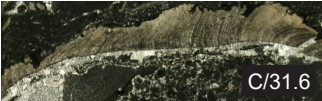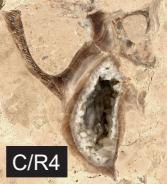

Supplement: Supplementary file 3 — Supplementary Figure S3. [file 41598_2021_99094_MOESM3_ESM.pdf]

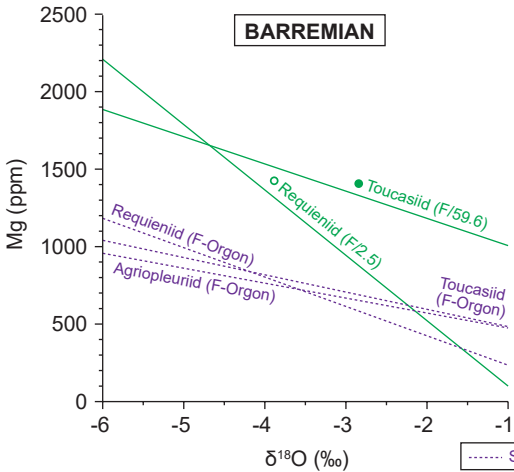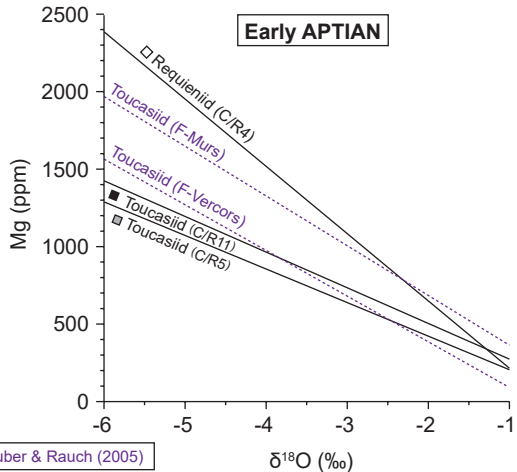

Supplement: Supplementary file 4 — Supplementary Figure S4. [file 41598_2021_99094_MOESM4_ESM.pdf]
