# Supplementary material for: Early Cretaceous sea surface temperature evolution in subtropical shallow seas
Source: Sci Rep. 2021 Oct 5;11:19765. doi: 10.1038/s41598-021-99094-2 (PMC8492702; doi:10.1038/s41598-021-99094-2)
Supplement: Supplementary file 6 — Supplementary Legends. [file 41598_2021_99094_MOESM6_ESM.pdf]

Figure S1 – Sclerochemistry and stratigraphy of rudists from France (Sausset section). Sclerochemical profiles ($\delta^{18}O$, $\delta^{13}C$) and calculated intra-annual SST variations (including seasonal mean values) of Barremian-Aptian rudist shells from France (Sausset section). Age assignments of shells are based on a rudist-shell carbon and strontium isotope stratigraphic framework, which allows for a correlation with pelagic carbon-isotope reference records (please refer to Figure 1). Polished slabs and microphotographs document the excellent preservation state of selected shells.

Figure S2 – Sclerochemistry and stratigraphy of rudists from France (Cluses section). Sclerochemical profiles ($\delta^{18}O$, $\delta^{13}C$) and calculated intra-annual SST variations (including seasonal mean values) of Upper Barremian rudist shells from France (Cluses section). Age assignments of shells are based on a (bulk carbonate) carbon and (rudist shell) strontium isotope stratigraphic framework, which allows for a correlation with pelagic carbon-isotope reference records (please refer to Figure 1). Both transmitted light and cathodoluminescence microphotographs document the pristine preservation state of selected shells.

Figure S3 – Sclerochemistry and stratigraphy of rudists from the Croatia (Kanfanar section). Sclerochemical profiles ($\delta^{18}O$, $\delta^{13}C$) and calculated intra-annual SST variations (including seasonal mean values) of Lower Aptian rudist shells from Croatia (Kanfanar section). Age assignments of shells are based on a (bulk carbonate and rudist shell) carbon and (rudist shell) strontium isotope stratigraphic framework, which allows for a correlation with pelagic carbon-isotope reference records (please refer to Figure 1). Polished slabs and microphotographs document the excellent preservation state of selected shells.

Figure S4 – Linear regressions of Mg/Ca vs. $\delta^{18}O$ data of Barremian-Aptian rudist bivalves.

Table S1 – Compilation of element and stable isotope sclerochemistry data. Element and stable isotope results and oxygen-isotope based sea surface temperatures (SSTs) of sclerochronological sampled rudist shells from Croatia, France, Spain and Portugal.
